# Dietary Animal Plasma Proteins Improve the Intestinal Immune Response in Senescent Mice

**DOI:** 10.3390/nu9121346

**Published:** 2017-12-11

**Authors:** Lluïsa Miró, Alba Garcia-Just, Concepció Amat, Javier Polo, Miquel Moretó, Anna Pérez-Bosque

**Affiliations:** 1 Departament de Bioquímica i Fisiologia, Facultat de Farmàcia i Ciències de l’Alimentació and Institut de Nutrició i Seguretat Alimentària, Universitat de Barcelona, 08028 Barcelona, Spain; lluisa.miro@ub.edu (L.M.); a.garcia.just@gmail.com (A.G.-J.); camat@ub.edu (C.A.); mmoreto@ub.edu (M.M.); 2 APC Europe S.L.U., 08403 Granollers, Spain; javier.polo@apc-europe.com

**Keywords:** intestinal inflammation, *S. aureus* enterotoxin B, aging, spray-dried animal plasma, functional proteins

## Abstract

Increased life expectancy has promoted research on healthy aging. Aging is accompanied by increased non-specific immune activation (inflammaging) which favors the appearance of several disorders. Here, we study whether dietary supplementation with spray-dried animal plasma (SDP), which has been shown to reduce the activation of gut-associated lymphoid tissue (GALT) in rodents challenged by *S. aureus* enterotoxin B (SEB), and can also prevent the effects of aging on immune system homeostasis. We first characterized GALT in a mouse model of accelerated senescence (SAMP8) at different ages (compared to mice resistant to accelerated senescence; SAMR1). Second, we analyzed the SDP effects on GALT response to an SEB challenge in SAMP8 mice. In GALT characterization, aging increased the cell number and the percentage of activated Th lymphocytes in mesenteric lymph nodes and Peyer’s patches (all, *p* < 0.05), as well as the expression of IL-6 and TNF-α in intestinal mucosa (both, *p* < 0.05). With respect to GALT response to the SEB challenge, young mice showed increased expression of intestinal IL-6 and TNF-α, as well as lymphocyte recruitment and activation (all, *p* < 0.05). However, the immune response of senescent mice to the SEB challenge was weak, since SEB did not change cell recruitment or the percentage of activated Th lymphocytes. Mice supplemented with SDP showed improved capacity to respond to the SEB challenge, similar to the response of the young mice. These results indicate that senescent mice have an impaired mucosal immune response characterized by unspecific GALT activation and a weak specific immune response. SDP supplementation reduces non-specific basal immune activation, allowing for the generation of specific responses.

## 1. Introduction

Aging is an intrinsic process that impairs the function of cells, tissue, organs, and individuals. It is characterized by an increased inflammatory state and physiological changes that involve a reduction in the response to environmental stimuli. These, in turn, increase predisposition to illness and death.

Francheschi et al. [1] introduced the concept of inflammaging, to define chronic low-grade inflammation that is shared by a broad spectrum of age-related pathologies. Inflammaging is caused by cumulative exposure to antigen loads, triggered by both clinical and subclinical infections, as well as exposure to non-infective antigens [2]. The resulting inflammatory condition involves the increased production of reactive oxygen species and pro-inflammatory cytokines, affecting both the innate [3] and acquired [4] immune system. This results in a vicious circle, which drives immune system remodeling and eventually favors a chronic pro-inflammatory state [5]. Hence, high plasma IL-6, IL-1, TNF-α, and C-reactive protein concentrations are associated with a higher risk of morbidity and mortality in elderly people [5].

The inflammaging profile is paralleled by changes in the immune system associated with aging that are known as immunosenescence. This is characterized by a decline in immune competence associated with a reduction in the secretion of specific IgA into the intestinal lumen, and a reduced capacity to generate tolerance to antigens [6]. This means that, in aged individuals, the protective function of gut-associated lymphoid tissue (GALT) is compromised.

In addition to progressive age-related GALT deterioration, the intestine is permanently exposed to food and environmental antigens, which are responsible for the permanent low-grade inflammation that characterizes the gut mucosa [7]. This condition can be reproduced in the laboratory by systemic administration of *Staphylococcus aureus* enterotoxin B (SEB), because it induces polyclonal activation of T cells, especially in the small intestine [8]. This results in mild stimulation of the secretion of pro-inflammatory cytokines [9,10,11].

Dietary intervention can modulate GALT activation. Dietary supplementation with functional proteins from porcine plasma (spray-dried plasma; SDP) has been shown to prevent the deterioration of the intestinal barrier and defense mechanisms, thereby reducing the degree of GALT activation in animals challenged by SEB [12,13]. The mechanism of action of SDP involves a reduction in the number of activated Th lymphocytes and stimulation of the Treg population, which restores the pro-/anti-inflammatory cytokine profile. This pattern has been extensively observed in the small intestine of rats [10] and mice [11].

Our aim here was to analyze whether SDP supplementation is effective in preventing the low-grade inflammation associated with senescence, and therefore whether supplemented animals are able to respond to an SEB challenge.

## 2. Materials and Methods 

### 2.1. Animals and Diets 

Male mice of the SAMP8 strain (prone to accelerated senescence) and its control senescence-resistant counterpart (SAMR1) [14] were obtained from Envigo (Bresso, Italy). The colonies generated were kept at the animal facility of the Faculty of Pharmacy and Food Science of the University of Barcelona under stable temperature and humidity conditions, with a 12 h:12 h light/dark cycle. All the protocols used in this study were approved by the Ethics Committee for Animal Use of the University of Barcelona and the Generalitat de Catalunya regional authorities (registration numbers 565/13 and 7397, respectively).

### 2.2. Experimental Design

Experiment 1. Characterization of GALT of SAMR1 and SAMP8 mice. In this experiment we used SAMP8 and SAMR1 mice at 2, 6, and 9 months of age. In these animals, the leukocyte populations in mesenteric lymph nodes (MLN) and Peyer’s patches (PP) were determined, as well as the expression of the pro-inflammatory cytokines *Il-6* and *Tnf-α* in the jejunum mucosa.

Experiment 2. Acute intestinal inflammation. In this experiment we used SAMP8 mice at 2 months of age (as young animals; 2M group), at 6 months of age (as reference senescent mice; 6M group), and at 6 months of age having been fed an SDP diet (as SDP senescent mice; 6M-SDP group). The mice received the experimental diets (Control diet and SDP diet; Table 1) from 2 to 6 months of age. Body weight evolution was recorded monthly and food consumption was recorded two times per week. Before SEB administration, blood was obtained by cheek puncture to measured plasma cytokines (IL-6, TNF-α). Intestinal inflammation was induced by an intraperitoneal administration of 25 µg SEB, while non-challenged mice received only the vehicle (phosphate buffered saline; PBS). All animals were killed by anesthetic overdose, 24 h after SEB or vehicle administration. In these animals were determined the leukocyte populations in MLN and PP, as well as the expression of the pro-inflammatory cytokines IL-6 and TNF-α in the jejunum mucosa.

### 2.3. MLN Cell Isolation 

Leukocytes from MLN were obtained as previously described [11]. Briefly, MLN were finely minced and incubated in a digestion solution composed of RPMI-1640 (Invitrogen, Carlsbad, CA, USA) with 5% inactivated fetal bovine serum (FBS), 100,000 U/L penicillin, 100 mg/L streptomycin, 10 mM HEPES, 2 nM L-glutamine, and 150 U/mL collagenase (Invitrogen, Carlsbad, CA, USA) at 37 °C in a shaker (Thermomixer Comfort Eppendorf^®^, Heuppauge, NY, USA). MLN were mechanically disaggregated and passed through a stainless-steel mesh. The cell suspension was centrifuged at 500× *g* for 10 min at 4 °C. The pelleted cells were resuspended in PBS-FBS. Cell number and viability were determined using acridine orange and ethidium bromide markers.

### 2.4. PP Cell Isolation 

Leukocytes from PP were obtained as previously described [9]. The PP were excised and incubated in the solution previously described for MLN. The PP were then mechanically disaggregated and passed through a stainless-steel mesh. The resulting cell suspensions were centrifuged at 500× *g* for 10 min at 4 °C. The pelleted cells were resuspended in 40% Percoll™ (GE Healthcare Bio-Sciences, Little Chalfont, Bucks, UK) in complete RPMI. This cell suspension was carefully layered over 80% Percoll™ in complete RPMI medium and centrifuged at 700× *g* for 20 min at 20 °C. The ring of cells was carefully collected, transferred to a clean tube, and centrifuged at 500× *g* for 10 min at 4 °C. The pelleted cells were resuspended in PBS-FBS. Cell number and viability were determined using acridine orange and ethidium bromide markers.

### 2.5. Cell Staining 

A total of 3 × 10^5^ cells were stained, as described previously by Pérez-Bosque et al. [9]. The conjugated primary antibodies used were anti-CD4 (GK1.5), anti-CD25 (PC61.5), and FoxP3 (FJK-16S), all obtained from eBioscience (San Diego, CA, USA). The stained cells were measured in a Gallios Flow cytometer (Beckman Coulter, Miami, FL, USA), located at the Cytometry Unit of the Scientific-Technical Services of the Barcelona Science Park. The results were analyzed using the Flowjo^®^ software (version 7.6.5, Treestar Inc., Ashland, OR, USA). Lymphocytes and non-lymphocytic leucocytes were separated by forward/side scatter.

### 2.6. Serum Cytokine Determination 

Serum concentration IL-6 and TNF-α were measured by Bio-Plex Cytokine Assay™ (Bio-Rad, Hercules, CA, USA).

### 2.7. Real-Time Polimerase Chain Reaction

RNA extraction, reverse transcription, and real-time polymerase chain reaction (PCR) were carried out as described previously by Pérez-Bosque et al. [11]. The primers used are shown in Table 2. The target gene transcripts were quantified using Glucuronidase β (*Gusβ*) gene expression as a reference, using the 2^−ΔΔCT^ method [15]. Product fidelity was confirmed by melt-curve analysis.

### 2.8. Statistical Analysis 

The results of the experiments are presented as the means ± standard error of the mean (SEM). The data were analyzed by two-way ANOVA followed by the Fisher’s least significant difference (LSD) post-hoc test, using GraphPad Prism^®^ software (version 6, GraphPad Software, Inc., La Jolla, CA, USA). In the first experiment, the factors considered were the strain of mice and age. In the second experiment, in body weight evolution and food consumption data the factors were time and diet; while in the immune variables the factors were age and dietary supplementation (considered together as a group variable: 2M, 6M, 6M-SDP), and SEB-induced inflammation. Plasma cytokines data was analyzed by one-way ANOVA followed by the Fisher’s LSD post-hoc test. Differences were considered significant at *p <* 0.05.

## 3. Results

### 3.1. Characterization of GALT in SAM Strains

#### 3.1.1. MLN Populations in SAM Strains

**Figure 1:**
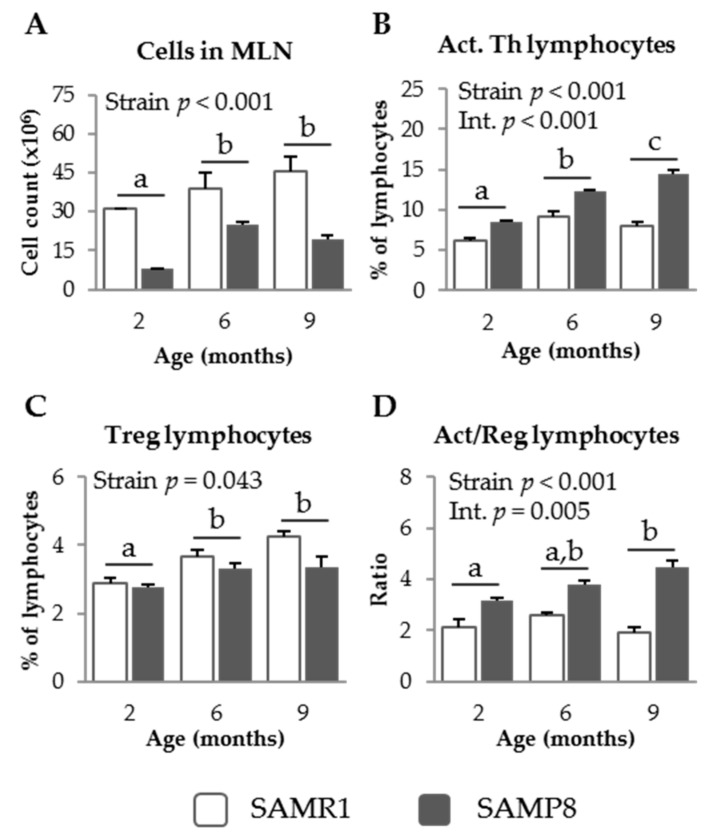
Cell recruitment (**A**); percentage of activated (Act.) Th lymphocytes (**B**); percentage of regulatory (Treg) Th lymphocytes (**C**); and the ratio of activated to regulatory Th lymphocytes (**D**), in mesenteric lymph nodes (MLN) of SAMR1 and SAMP8 mice at 2, 6, and 9 months of age. The open bars represent SAMR1 mice; the solid bars, SAMP8 mice. Results are expressed as means ± SEM (*n* = 5–11 animals). Means without a common letter differ, *p* < 0.05. Int., interaction between factors (age and mice strain).

SAMP8 mice showed lower MLN leukocyte counts than the SAMR1 strain at all of the ages studied (*p* = 0.001, Figure 1A). The percentage of activated Th lymphocytes in SAMP8 MLN was higher than that in SAMR1 (*p* < 0.001, Figure 1B), and this effect was enhanced in the older mice (interaction; *p* < 0.001). In contrast, SAMR1 mice showed higher percentages of regulatory Th lymphocytes than SAMP8 mice (*p* = 0.043, Figure 1C). Moreover, the percentage of this population increased with age (*p* = 0.003). The ratio of activated to regulatory Th lymphocytes in MLN was significantly higher in SAMP8 than in SAMR1 mice (*p* < 0.001, Figure 1D) and this effect was more evident at 9 months of age, indicating a significant interaction between mouse strain and age (*p* = 0.005).

#### 3.1.2. PP Populations in SAM Strains

**Figure 2:**
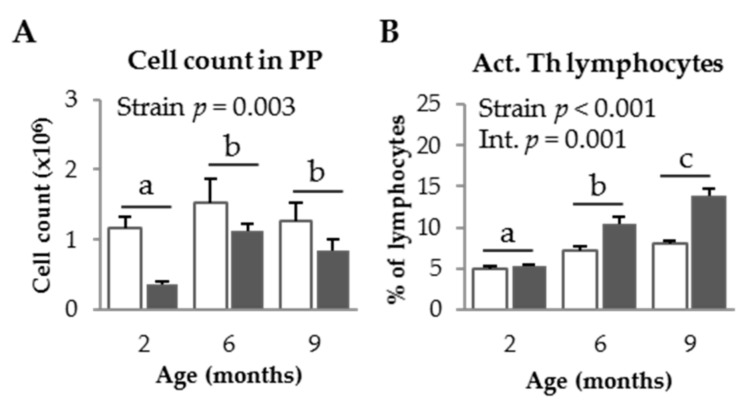

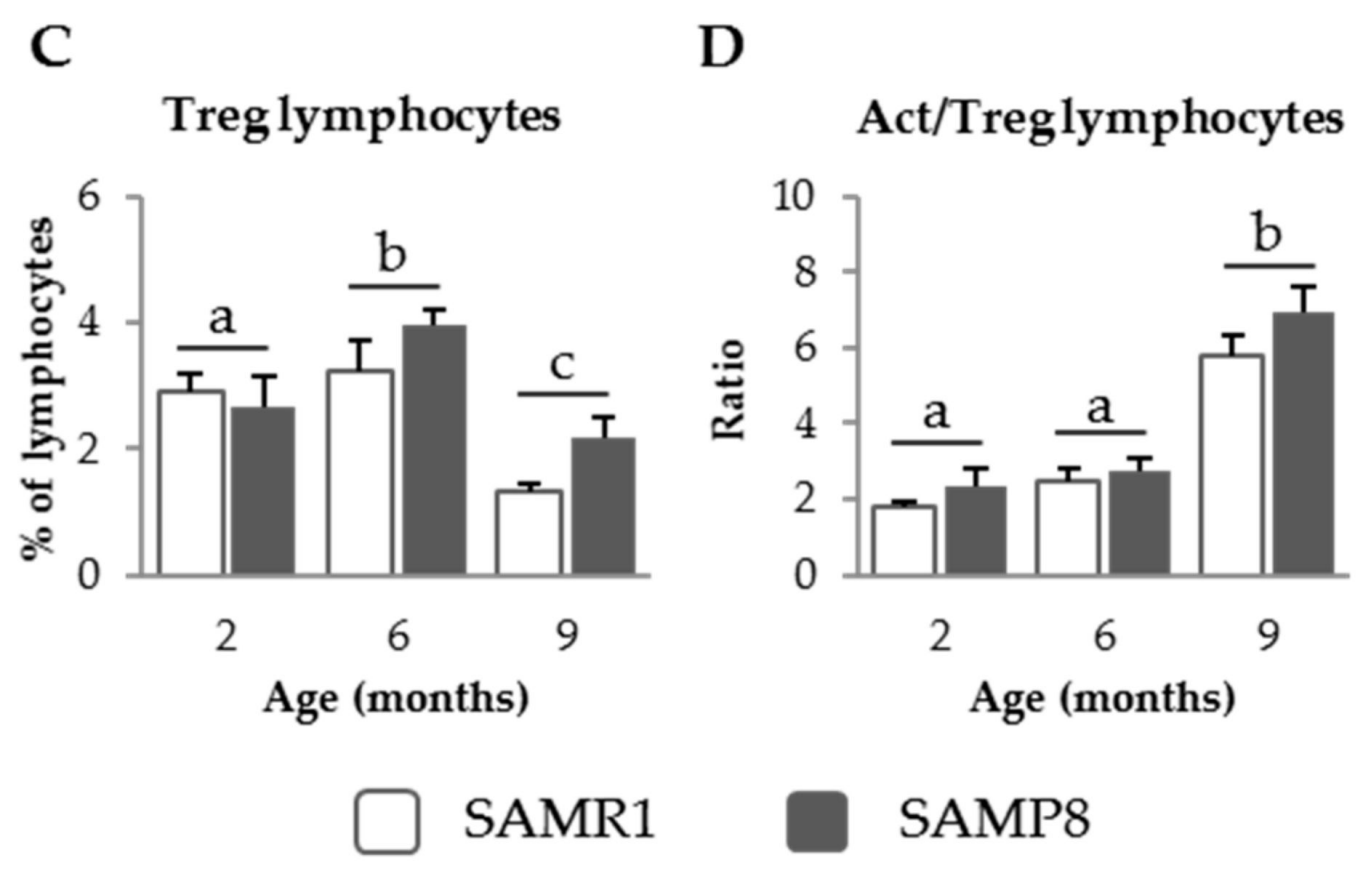
Cell recruitment (**A**); percentage of activated (Act.) Th lymphocytes (**B**); percentage of regulatory (Treg) Th lymphocytes (**C**); and the ratio of activated to regulatory Th lymphocytes (**D**), in Peyer’s patches (PP) of SAMR1 and SAMP8 mice at 2, 6, and 9 months of age. The open bars represent SAMR1 mice; the solid bars, SAMP8 mice. Results are expressed as means ± SEM (*n* = 5–10 animals). Means without a common letter differ, *p* < 0.05. Int., interaction between factors (age and mice strain).

As for MLN, the PP cell count was higher in SAMR1 mice than in SAMP8 mice (*p* = 0.003, Figure 2A), and increased with age in both mice strains (*p* = 0.047). The percentage of activated Th lymphocytes was higher in SAMP8 mice than in SAMR1 mice (*p* < 0.001, Figure 2B) and also increased with age (*p* < 0.001). Moreover, the effect of age was more pronounced in SAMP8 mice than in SAMR1 mice (*p* = 0.001). The percentage of regulatory Th lymphocytes decreased in mice from both strains at 9 months of age (*p* < 0.001; Figure 2C). The ratio of activated to regulatory Th lymphocytes increased with age (*p* < 0.001, Figure 2D).

#### 3.1.3. Pro-Inflammatory Cytokines in SAM Strains

**Figure 3:**
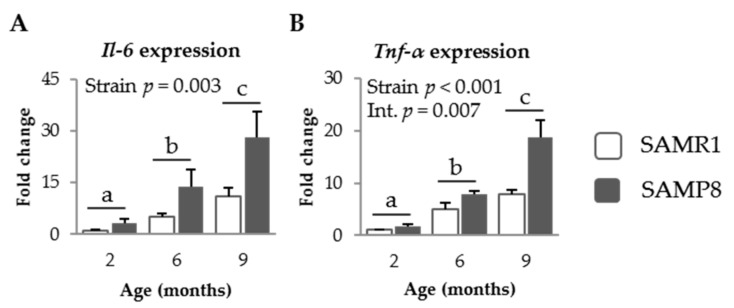
mRNA expression of *Il-6* (**A**) and *Tnf-α* (**B**), in the jejunum mucosa of SAMR1 and SAMP8 mice at 2, 6, and 9 months of age. The open bars represent SAMR1 mice; the solid bars, SAMP8 mice. All the target genes were normalized to glucuronidase β (*Gusβ*) expression. The fold change expression is related to SAMR1 mice of 2 months of age. Results are expressed as means ± SEM (*n* = 6–7 animals). Means without a common letter differ, *p* < 0.05. Int., interaction between factors (age and mice strain).

SAMP8 mice showed higher expression levels of *Il-6* and *Tnf-α* in their jejunum mucosa than SAMR1 mice (*p* = 0.003 and *p* < 0.001, respectively, Figure 3A,B). Aging increased the intestinal expression of both cytokines in both mice strains (*p* = 0.001 and *p* < 0.001, respectively), although SAMP8 mice exhibited a more evident effect of age on expression of *Tnf-α* than SAMR1 mice (*p* = 0.007).

### 3.2. Effect of SDP on Senescent Mice

#### 3.2.1. Effect of SDP on Systemic Variables

**Figure 4:**
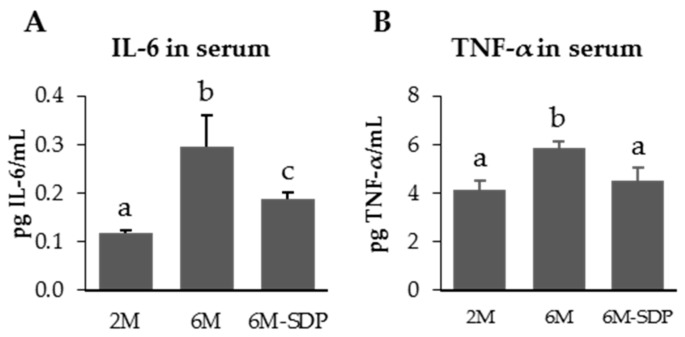
Serum concentration of IL-6 (**A**) and TNF-α (**B**) of SAMP8 mice of the 2-month-old (2M), 6-month-old (6M), and 6-month-old fed spray-dried plasma (6M-SDP) groups. Results are expressed as means ± SEM (*n* = 6–7 animals). Means without a common letter differ, *p* < 0.05.

SDP supplementation did not modify body weight evolution nor food consumption (Figure S1). The concentration of pro-inflammatory cytokines IL-6 and TNF-α increased in the serum of senescent mice (*p* < 0.001 and *p* = 0.011; Figure 4), and SDP supplementation reduced both cytokines in aged mice (*p* = 0.046 and *p* = 0.041, respectively).

#### 3.2.2. Effect of SDP on MLN Populations in Senescent Mice

**Figure 5:**
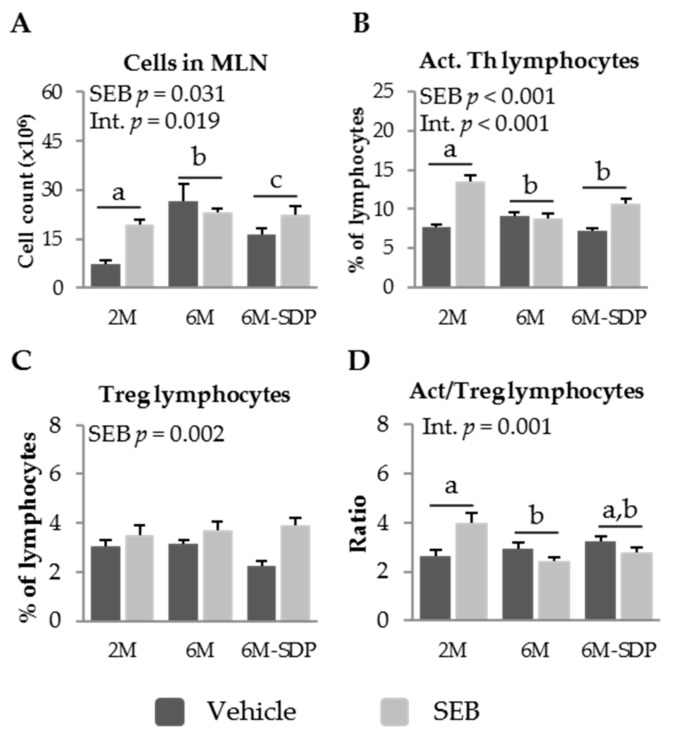
Cell recruitment (**A**); percentage of activated (Act.) Th lymphocytes (**B**); percentage of regulatory (Treg) Th lymphocytes (**C**); and the ratio of activated to regulatory Th lymphocytes (**D**), in mesenteric lymph nodes (MLN) of SAMP8 mice of the 2-month-old (2M), 6-month-old (6M), and 6-month-old fed spray-dried plasma (6M-SDP). The dark grey bars represent groups that received the vehicle and the light grey bars represent mice administered with *S. aureus* enterotoxin B (SEB). Results are expressed as means ± SEM (*n* = 5–8 animals). Means without a common letter differ, *p* < 0.05. Int., interaction between factors (SEB administration and experimental groups; 2M, 6M, and 6M-SDP).

As stated for the previous result, senescent mice (6 months old) showed increased cell recruitment into MLN under basal conditions (*p* < 0.001), which was attenuated by SDP supplementation (*p* = 0.043). SEB administration increased cell recruitment into MLN (*p* = 0.031, Figure 5A), although senescent mice fed the control diet showed a weaker response to SEB administration than young mice or senescent mice supplemented with SDP (*p* = 0.019). SEB administration increased the percentage of activated Th lymphocytes (*p* < 0.001; Figure 5B). SEB had no effect on aged mice (6M) fed the control diet, while young SAMP8 and SDP-supplemented old mice responded to administration of the toxin (*p <* 0.001). Neither senescence nor dietary supplementation with SDP modified the percentage of regulatory Th lymphocytes in older compared to younger SAMP8 mice (Figure 5C). In contrast, SEB administration increased the percentage of regulatory Th lymphocytes in all groups (*p* = 0.002). The ratio of activated to regulatory Th lymphocytes was lower in aged mice, suggesting a reduced immune response to SEB (*p* = 0.012, Figure 5D). SDP supplementation did not modify the ratio of activated to regulatory Th lymphocytes observed in 6-month-old mice. Moreover, there was an interaction between age and SEB immune response (*p* = 0.001), since SEB increased this ratio in young mice, while in senescent mice the ratio was reduced.

#### 3.2.3. Effect of SDP on PP Populations in Senescent Mice

**Figure 6:**
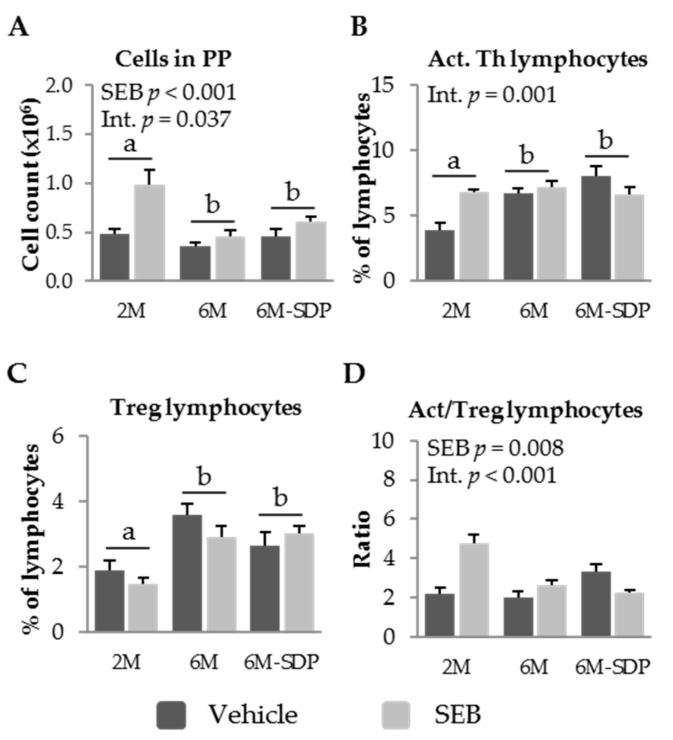
Cell recruitment (**A**); percentage of activated (Act.) Th lymphocytes (**B**); percentage of regulatory (Treg) Th lymphocytes (**C**); and the ratio of activated to regulatory Th lymphocytes (**D**), in Peyer’s patches (PP) of SAMP8 mice of the 2-month-old (2M), 6-month-old (6M), and 6-month-old fed spray-dried plasma (6M-SDP). The dark grey bars represent groups that received the vehicle and the light grey bars represent mice administered with *S. aureus* enterotoxin B (SEB). Results are expressed as means ± SEM (*n* = 6–8 animals). Means without a common letter differ, *p* < 0.05. Int., interaction between factors (SEB administration and experimental groups; 2M, 6M, and 6M-SDP).

SEB administration increased leukocyte recruitment into PP in all of the experimental groups (*p* < 0.001; Figure 6A). The percentage of activated Th lymphocytes in PP was higher in senescent animals than in young animals (*p* = 0.015; Figure 6B). SDP supplementation did not affect this variable. Though no clear effects of SEB were observed, there was an interaction between SEB administration and SDP supplementation (*p* = 0.001). Senescence was characterized by an increase in the percentage of regulatory Th lymphocytes in PP (*p* < 0.001; Figure 6C). SDP supplementation did not attenuate the effects of senescence on the percentage of regulatory Th lymphocytes in PP, and SEB administration did not modify this population. The administration of the enterotoxin generated different responses in the ratio between activated and regulatory Th cells in PP in all of the groups (SEB effect *p* = 0.008, Figure 6D). SEB administration increased this ratio in young (2-month-old) and aged (6-month-old) mice fed the control diet. Moreover, the enterotoxin reduced the activation to regulatory lymphocyte ratio in the group that received the SDP supplementation. There was an interaction between SEB administration and SDP supplementation (*p* < 0.001).

#### 3.2.4. Effect of SDP on Cytokine Expression in Senescent Mice

**Figure 7:**
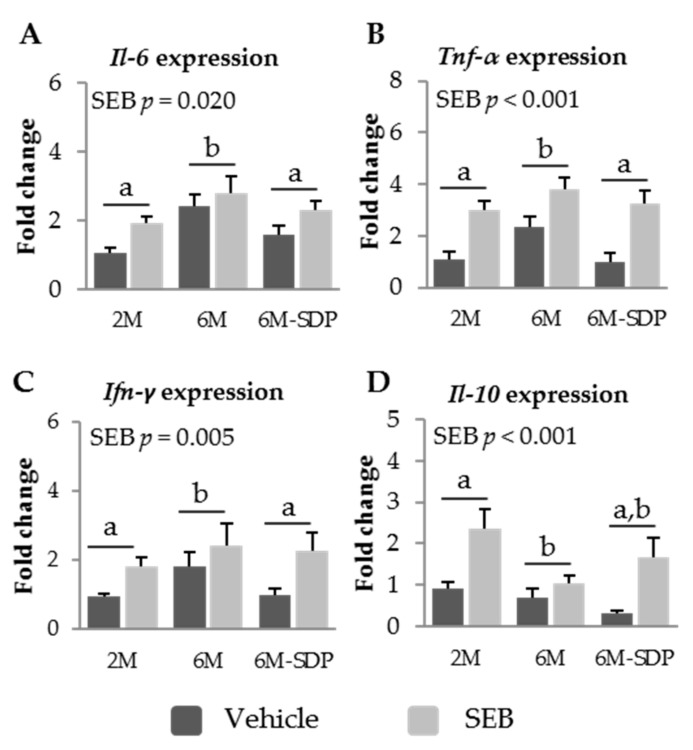
mRNA expression of *Il-6* (**A**), *Tnf-α* (**B**), *Ifn-γ* (**C**), and *Il-10* (**D**) in the jejunum mucosa of SAMP8 mice of the 2-month-old (2M), 6-month-old (6M), and 6-month-old fed spray-dried plasma (6M-SDP). The dark grey bars represent groups that received the vehicle and the light grey bars represent mice administered with *S. aureus* enterotoxin B (SEB). All the target genes were normalized to glucuronidase β (*Gusβ*) expression. The fold change expression is related to 2M mice administered with vehicle. Results are expressed as means ± SEM (*n* = 5–6 animals). Means without a common letter differ, *p* < 0.05. Int., interaction between factors (SEB administration and experimental groups; 2M, 6M, and 6M-SDP).

Senescence increased the expression of *Il-6*, *Tnf-α*, and *Ifn-γ* in the mucosa of the jejunum (*p* = 0.002, *p* = 0.019, and *p* = 0.006, respectively, Figure 7A–C). This effect was prevented by SDP supplementation (*p* = 0.040 for *Il-6*; *p* = 0.020 for *Tnf-α*; and *p* = 0.026 for *Ifn-γ*). Moreover, SEB administration increased the expression of the three cytokines (*p* = 0.020 for *Il-6*, *p* < 0.001 for *Tnf-α*; and *p* = 0.009 for *Ifn-γ*). Administration of the toxin increased the expression of the anti-inflammatory cytokine *Il-10* in the jejunum mucosa (*p* < 0.001; Figure 7D). However, senescence impaired the mucosal expression of this cytokine during the acute inflammation induced by the SEB challenge (*p* = 0.021).

## 4. Discussion

Senescence is characterized by an increase in the basal inflammatory state, which contributes to the development and progression of various diseases associated with aging. This increase in the inflammatory state leads to a decrease in the functionality of the immune system and a reduced capacity to respond to the presence of infections or external aggressions, known as immunosenescence [16]. In addition, this decrease is characterized by low-intensity inflammation sustained over time, known as inflammaging, with increased production of pro-inflammatory cytokines and other mediators of inflammation [2,17]. In aged individuals, the gastrointestinal tract is particularly susceptible to infectious diseases, and this is associated with a lower capacity to respond of the mucosal immune system [18]. Therefore, more in-depth knowledge of the alterations that occur during the aging of the immune system would allow us to design strategies to delay immunosenescence and decrease the incidence of associated pathologies.

We used SAMP8 mice, which are widely used in aging studies, because this strain exhibits morphological and functional alterations associated with senescence, already at early stages of development [19]. SAMP8 mice show normal growth and development for the first 2 months of life, after which they show changes in the immune system associated with aging, such as deterioration in the capacity of Th lymphocytes to respond [20]. Our results showed that young SAMR1 and SAMP8 mice have similar immune characteristics, although SAMP8 mice have lower lymphocyte counts in both MLN and PP than SAMR1 mice, and these differences become larger with age.

Aging alters the production of cytokines, which in turn modulates the immune system as cytokines regulate the growth and differentiation of leukocytes and exhibit numerous biological activities [21]. Several studies have shown that aging is accompanied by an increase in the production of pro-inflammatory cytokines, such as IL-6, IL-1β, and TNF-α, which promote chronic inflammation in the elderly [15,22] and favor the onset and progression of degenerative diseases that are common in this period [4]. At the same time, increased pro-inflammatory cytokine concentrations in the intestinal mucosa compromise the integrity and permeability of the epithelium [23]. We observed that aged mice secrete increased amounts of the pro-inflammatory cytokines *Il-6* and *Tnf-α* from the jejunum mucosa and that this effect is more pronounced in SAMP8 mice than in the SAMR1 mice. This effect is accompanied by an increase in the percentage of activated Th lymphocytes from the gut-associated immune system that is already visible in 6-month-old animals. 

One of the most remarkable alterations that is observed in aging is a decline in the performance of adaptive immunity mechanisms and the establishment of a mild chronic inflammatory state. With this in mind, chronic antigenic load and inflammaging are the major causes of aging and its related diseases [24]. Our current data show that 6-month-old SAMP8 mice already present an immunosenescence profile as well as inflammaging characteristics typical of old animals, and that these processes have already begun at 2 months of age. Based on these results, the intestinal immune response of SAMP8 mice to SEB and the effect of dietary supplementation with SDP were studied.

Elderly people have a decreased capacity to generate adequate adaptive responses, making them more susceptible to systemic infections and side effects of medication [25]. Aging is characterized by a loss of the responsiveness of T cells [16] and altered function of Treg lymphocytes which makes them less efficient at inhibiting the pro-inflammatory activities of other cell types [26,27]. Thus, Treg lymphocytes from aged individuals are less efficient at preventing inflammaging, although their numbers increase with age in lymphoid tissues. Several studies in mice have demonstrated a greater presence of Treg lymphocytes in the lymphoid organs of old animals than those of young animals, while the number in circulating blood remains unchanged [26,28]. Our results demonstrate that this phenomenon also occurs in the MLN of SAMP8 and SAMR1 mice.

Under basal conditions, we observed that dietary supplementation of 6-month-old SAMP8 mice with SDP prevents the progression of intestinal inflammation, by reducing the expression of the pro-inflammatory cytokines *Il-6*, *Tnf-α*, and *Ifn-γ*. Moreover, aged mice also had increased serum concentration of TNF-α and IL-6, thus confirming observations made by Brüünsgaard and Pedersen [5]. The increased plasma concentration of pro-inflammatory cytokines supports the view that aging is characterized by an inflammatory profile [2]. Dietary SDP supplementation reduced the serum concentration of pro-inflammatory cytokines, which indicates that SDP reduce the inflammaging profile observed in aged mice.

Activation of the immune system with superantigens such as SEB leads to increased production of the pro-inflammatory cytokines TNF-α, IFN-γ, and IL-2 [29]. Our results also showed that 6-month-old SAMP8 mice have low responsiveness to SEB because no increase in *Il-6* or *Ifn-γ* cytokine expression was observed. In contrast, the intestinal mucosa of 2-month-old mice, as well as mice fed SDP, showed an increased production of pro-inflammatory cytokines after SEB administration, as well as an increase in the anti-inflammatory cytokine *Il-10*. Our results also indicate that aged SAMP8 mice have an impaired immune response to SEB, since the number of lymphocytes in GALT remains unchanged after its administration and the activated Th lymphocyte subpopulation was not increased. In young SAMP8 mice, however, there was an increase in the recruitment and activation of Th lymphocytes, in both MLN and PP, indicating that the immune system responds to the toxin. The mice supplemented with SDP increased the recruitment of lymphocytes in both MLN and PP, as well as the percentage of activated Th cells in MLN, in response to the enterotoxin in aged mice.

Therefore, although inflammaging results in a large number of alterations at advanced ages, such as cardiovascular disease or chronic obstructive pulmonary disease [1,2,30], and contributes to an insufficient response to infections, it does not result in an increased immune response [31,32]. Rather, the data suggest a failure to generate a specific immune response, as well as a subsequent inability to resolve the inflammation. Our current results indicate that dietary supplementation with SDP minimizes the loss of the capacity to respond to an infection that occurs in the intestine of senescent mice; SDP decreases inflammaging and allows a proper response to an inflammatory stimulus.

Changes in the composition of the gut microbiota throughout aging likely contribute to immunosenescence and to the development of a pro-inflammatory phenotype [33]. There is consistent evidence supporting the view that SDP supplements can modify the intestinal microbiota. For example, Torrallardona et al. [34] reported that porcine SDP favors the growth of lactobacilli in the ileum and cecum of pigs. In rodents, we observed that SDP can increase the frequency of detection of several species of *Lactobacillus* in the rat cecum [35] as well as in fecal samples from SAM mice [36]. Preliminary studies performed in young mice showed that SDP supplementation favors the growth of bacterial families that are butyrate and acetate producers [37]. These results suggest that some components in SDP may be effective in changing the profile of the microbiota, with potential implications for reducing intestinal and peripheral inflammation.

The effects of dietary supplementation with SDP on GALT have previously been studied in young animals, but there were no studies of its effects on old animals. Pérez-Bosque et al. [11] found that SDP has a considerable anti-inflammatory effect after an SEB challenge in young mice, thus averting an exaggerated reaction by the immune system to the toxin. Several studies of the mechanism by which dietary SDP modulates the GALT immune system suggest that it decreases mucosal pro-inflammatory cytokine expression, reduces the activation of Th lymphocytes, and promotes an abundance of regulatory Th lymphocytes. In addition, dietary SDP also increases the production of anti-inflammatory cytokines such as IL-10 or TGF-β, which counteract the effects of TNF-α and other mediators of inflammation. These changes in the cytokine profile may explain the reduced effects of SEB administration on young mice [11]. In this study, we observed that 6-month-old SAMP8 mice that received dietary SDP supplementation presented changes in their cytokine profile. The pro-inflammatory cytokines *Il-6*, *Tnf-α*, and *Ifn-γ* were diminished compared to the aged group, which would explain the prevention of inflammaging. In addition, SDP decreases the subpopulation of activated Th lymphocytes in MLN. This decrease in basal inflammation in aged mice is key in generating a response of the immune system to other stimuli, such as SEB administration. In this way, the mice that received the SDP feed can respond in a similar way to the younger mice.

## 5. Conclusions

Senescent mice have an impaired mucosal immune response characterized by unspecific GALT activation and a weak specific immune response. SDP exerts a modulatory effect on the intestinal immune system because it decreases basal inflammation associated with age (inflammaging) by promoting mucosal Treg lymphocytes and IL-10 production. This allows an effective immune response when mice are challenged with SEB.

## Figures and Tables

**Table 1:** Composition of experimental diets.

Ingredients	Control Diet	SDP ^1^ Diet
	g/kg	
SDP	-	80.0
Dried skim milk	530.7	340.5
Corn starch	199.3	308.8
Sucrose	94.5	94.5
Soybean oil	70	70
Cellulose	50	50
AIN-93-G-MX (94046) ^2^	35	35
AIN-93 VX (94047) ^2^	15	15
DL-Methionine	2.5	3.2
Choline bitartrate	3	3

^1^ SDP (spray-dried plasma) was provided by APC Europe, S.L.U., Granollers, Spain; ^2^ AIN-93 VX, vitamin mix; AIN-93-G-MX, mineral mix. Both were provided by Envigo, Bresso, Italy.

**Table 2:** Primers used for real-time PCR.

Primer	Forward (5′-3′)	Reverse (5′-3′)	Fragment Size	Accession Number
*Gusβ*	CCGATTATCCAGAGCGAGTATG	CTCAGCGGTGACTGGTTCG	197 bp	NM_010368.1
*Il-6*	ACCAGAGGAAATTTTCAATAGGC	TGATGCACTTGCAGAAAACA	109 bp	NM_031168.1
*Il-10*	GGCGCTGTCATCGATTTCTCCCC	TGGCCTTGTAGACACCTTGGTCTT	102 bp	NM_010548.2
*Inf-γ*	CCTTCTTCAGCAACAGCAAGGCG	CTTGGCGCTGGACCTGTGGG	87 bp	NM_008337.4
*Tnf-α*	CCACCACGCTCTTCTGTCTAC	AGGGTCTGGGCCATAGAACT	103 bp	NM_013693.2

*Gusβ*, glucuronidase β; *Il-6*, Interleukin 6; *Il-10*; interleukin 10; *Inf-γ*, interferon-γ; *Tnf-α*, tumoral necrosis factor α.

## Supplementary Materials

The following are available online at www.mdpi.com/2072-6643/9/12/1346/s1, Figure S1: Body weight evolution (A) and food consumption (B) of SAMP8. The body weight of SAMP8 mice was measured once a month. Food intake was measured three times per week during the feeding period. Results are expressed as means ± SEM (*n* = 6–8 mice).
