# Supplementary material for: Dietary Animal Plasma Proteins Improve the Intestinal Immune Response in Senescent Mice
Source: Nutrients. 2017 Dec 11;9(12):1346. doi: 10.3390/nu9121346 (PMC5748796; doi:10.3390/nu9121346)
Supplement: Supplementary file 1 [file nutrients-09-01346-s001.zip › nutrients-247589-supplementary.pdf]

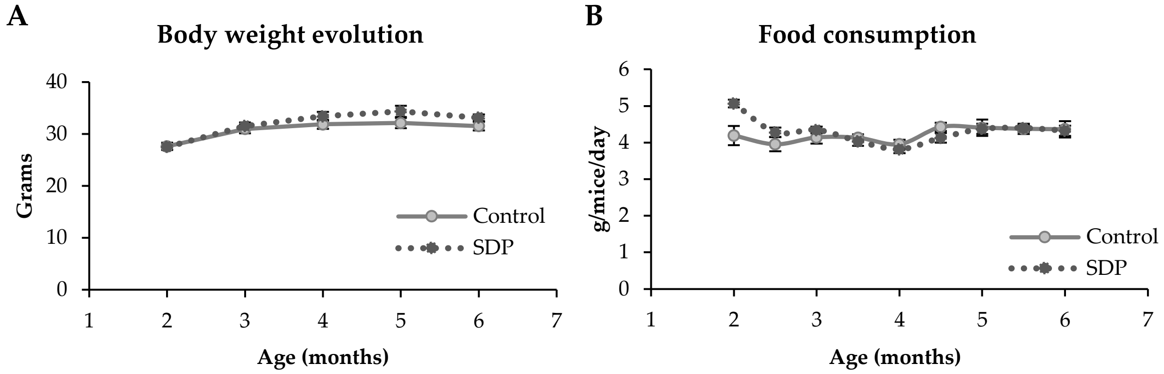


Figure S1. Body weight evolution (A) and food consumption (B) of SAMP8. The body weight of SAMP8 mice has been measured once a month. Food intake has been measured three times per week during the feeding period. Results are expressed as mean ± SEM (n=6-8 mice).
